# Recent Developments in High-Performance Membranes for CO_2_ Separation

**DOI:** 10.3390/membranes11020156

**Published:** 2021-02-23

**Authors:** Zi Tong, Ali K. Sekizkardes

**Affiliations:** 1 National Energy Technology Laboratory, U.S. Department of Energy, 626 Cochrans Mill Road, Pittsburgh, PA 15236, USA; 2 Oak Ridge Institute for Science and Education, Pittsburgh, PA 15236, USA; 3 Leidos Research Support Team, 626 Cochrans Mill Road, P.O. Box 10940, Pittsburgh, PA 15236, USA

## Abstract

In this perspective article, we provide a detailed outlook on recent developments of high-performance membranes used in CO_2_ separation applications. A wide range of membrane materials including polymers of intrinsic microporosity, thermally rearranged polymers, metal–organic framework membranes, poly ionic liquid membranes, and facilitated transport membranes were surveyed from the recent literature. In addition, mixed matrix and polymer blend membranes were covered. The CO_2_ separation performance, as well as other membrane properties such as film flexibility, processibility, aging, and plasticization, were analyzed.

## 1. Introduction

Despite the remarkable renewable energy capacities being operated around the world, global CO_2_ concentrations continue to rise. A set of transformational carbon capture, utilization, and storage (CCUS) technologies are urgently needed that can prevent CO_2_ from entering the atmosphere, use the captured CO_2_ for downstream applications, and safely and permanently store it deep under the ground [1]. Membrane gas separation technology has been believed to be one of the most promising technologies to replace the traditional technologies such as amine scrubbing, due to its small footprint, simplicity, and high energy efficiency. To overcome the challenges in lowering carbon capture cost or profitable carbon capture, a membrane material will need to be on or above the Robeson Upper Bound, i.e., in the high permeability/moderate selectivity regime [2]. The state-of-the-art membranes utilized in industry are heavily dependent on conventional membrane materials such as cellulose acetate, polysulfone, polydimethylsiloxane, polyethylene oxide, etc. Applications of these gas separation membranes are clustered around hydrogen recovery and nitrogen separation, methane reforming, and vapor recovery [3]. On the other hand, current CO_2_ separation membranes have been relatively less preferred and did not surpass the cost efficiency of conventional CO_2_ separation technologies such as solvents and sorbents. Recent studies suggested that the CO_2_ permeability for a potential membrane candidate should be at least two orders of magnitude larger than the permeability of conventional membranes to break through [4]. The high CO_2_ permeability should be also complemented with adequate selectivity over other gases, such as nitrogen, in gas mixtures. Therefore, there is a clear need for more advanced membrane materials to be used CO_2_ separation. Recently, a growing amount of interest has been concentrated in advanced membrane materials to achieve cost-efficient CO_2_ separation. A large body of literature has been accumulated in the past decade covering these high-performance membranes. Accordingly, several new classes of advanced membrane materials have been introduced. Membranes include polymers of intrinsic micro porosity (PIMs) [5], thermally rearranged (TR) polymers [6], polyethylene oxide (PEO)-based copolymers [7], poly ionic liquids [8] and facilitated transport membranes [9]. In addition, mixed matrix and polymer blend membranes have been introduced by mixing membranes with versatile types of filler materials and polymers [10]. Although most of the membranes are polymeric materials, more recently, promising results have been also reported for inorganic membranes using metal–organic frameworks [11].

## 2. Discussion

**Figure 1:**
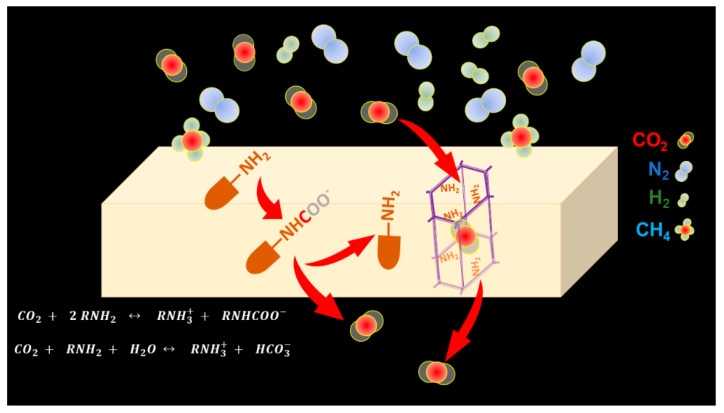
Schematic of CO_2_ and amine carrier interaction and an ultrafast CO_2_-selective transport channel inside a facilitated transport membrane.

Facilitated transport membranes are an emerging solution that combines the reversible reaction between CO_2_ and the carrier with the intrinsic kinetic properties of a membrane process. On the feed interface of the membrane, CO_2_ reacts with a free amine and forms a carbamate complex, which diffuses through the membrane into the permeate interface of the membrane (Figure 1). With the presence of inert sweeping gas or vacuum on the permeate side, CO_2_ dissociates from the complex because of the near-zero CO_2_ partial pressure on the permeate side. Meanwhile, the non-reactive gases, such as N_2_, H_2_, and CH_4_, can only transport through the membrane via a physical solution–diffusion process, in which the gas is adsorbed by the membrane on the feed interface and then diffuses through the membrane along its concentration gradient to the permeate interface where the desorption occurs. Most non-facilitated polymeric membranes separate gases following the solution–diffusion mechanism, based on the differences in solubility and/or diffusivity of gas pairs. A variety of approaches are being explored and studied to improve the permeability and selectivity of facilitated transport membranes, such as tuning the chain flexibility of the hydrogel polymer, increasing the carrier density inside the membrane, etc. This article focuses on the discussion of two highly effective approaches, which represent the most promising future direction of the technology: (1) improving the carrier efficiency by tuning the molecular structure and (2) constructing the ultrafast CO_2_-selective transport channel.

As a result of the specific reaction between CO_2_ and the amine carrier, facilitated transport membranes usually possess high selectivity. However, the amine is also the origin of a hydrogen bonding formation that results in a reduction in the diffusivity of the CO_2_–amine complex. To disrupt the hydrogen-bonding network, a favorable carrier molecular structure is needed. The Hideto Matsuyama research group at Kobe University investigated a variety of amino acid ionic liquid-type carriers with different molecular structures and found that a cyclic amino acid ionic liquid had the smallest viscosity increase upon CO_2_ absorption owing to the ring structure that limits the available hydrogen atoms to form hydrogen bonding [12]. They later used the prolinate ionic liquid carrier and achieved an ultra-high CO_2_ permeability of 52,000 Barrer and CO_2_/N_2_ selectivity of 8100 at 30 °C, 70% relative humidity (RH) and 0.1 KPa of CO_2_ partial pressure, showing great promise for the direct capture of CO_2_ from the atmosphere [13].

The above finding of cyclic amino acid ionic liquid also reveals a fact that the steric hindrance of an amino group can interfere with the hydrogen bonding formation. What is more, according to the CO_2_–amine chemistry, a sterically hindered amine can augment the CO_2_-loading capacity because of the difficulty of forming a carbamate complex, thus changing the stoichiometry of the overall CO_2_–amine reaction. By attaching a bulky group to the amine, scientists can manipulate the steric hindrance of the amine carriers. Ho et al. for the first time introduced the amine steric hindrance effect previously applied on solvent absorption into the solid facilitated transport membrane via the attachment of an isopropyl group to the amino groups of polyallylamine [14]. Considering that polyallylamine is less favorable than polyvinylamine in terms of film-forming ability and CO_2_ separation performance, Tong and Ho later modified polyvinylamine with methyl groups and significantly improved the CO_2_/H_2_ separation performance by three times at 102 °C [15]. In the Ho group’s most recent work, they deprotonated a series of α-aminoacids with different alkyl or hydroxyethyl substituents by 2-(1-piperazinyl)ethylamine, leading to the nonvolatile amine carriers with different degrees of steric hindrance. A remarkably high CO_2_ permeance of 435 GPU (gas permeation units) along with unprecedented CO_2_/H_2_ selectivity of over 500 was achieved at 107 °C and 0.4 atm of CO_2_ partial pressure, which is well above the Robeson Upper Bound, which is an empirical limit describing the trade-off between permeability and selectivity of polymer membranes.

In addition to the efforts of tuning the amine structure to achieve better separation performance, it is noteworthy that the construction of ultrafast CO_2_ transport channels in facilitated transport membranes significantly advances this field as well. This is a relatively unexplored area in the facilitated transport membrane literature. Since the porous fillers often come with the drawback of low selectivity, they can negate the most attractive advantage of facilitated transport membrane (i.e., the high selectivity). Another limitation is the poor compatibility of mostly hydrophobic porous fillers with hydrophilic amine materials. Recently though, there have been a few advances in this area. Decoration of those metal–organic frameworks (MOFs) and covalent organic frameworks (COFs) with amine-containing functional groups can achieve preferable CO_2_ adsorption and good compatibility with the polyamine matrix at the same time. The Zhi Wang research group at Tianjin University put forward a strategy for in situ synthesized polyethyleneimine grafted zeolitic imidazolate frameworks ZIF-8. The redundancy of amino groups in polyethyleneimine (PEI) enabled it to coordinatively react with Zn^2+^ similar to imidazole, thus grafting it into the crystal structure of ZIF-8. Then, the PEI-g-ZIF-8, with an average pore size of 7.53 Å (CO_2_ kinetic diameter: 3.3 Å), was dispersed into polyvinylamine and formed a gas transport channel-embedded facilitated transport membrane. By this approach, they obtained CO_2_ permeance of 1990 GPU and CO_2_/N_2_ selectivity of 79.9 for a typical flue gas carbon capture scenario [16]. The idea of grafting polyamine onto organic frameworks was further extended by the Wang group with the development of a COF/polymer hybrid membrane by penetrating polyvinylamine into the oversized pore (1.8 nm) of a two-dimensional COF constructed of triformylbenzene and diaminobenzene. As a result of the reaction between the amino groups of polyvinylamine and the pendant formyl groups on the COF pore walls, part of polyvinylamine was chemically immobilized onto the COF, resulting in a membrane with well-dispersed COFs in the polyvinylamine matrix with good interfacial compatibility. The amino environment of the COF pore walls favored the adsorption–diffusion of CO_2_ through the pores while excluding other gases such as N_2_, CH_4_, and H_2_ [17]. Recently, a gravity-induced interface self-assembly approach was reported by the Wang group to construct an ultrafast transport channel in facilitated transport membranes [18]. In this work, they innovatively leveraged the different affinities of modified MIL-101(Cr) and polyvinylamine onto the membrane substrate and formed well-dispersed modified MIL-101 nanoparticles in the polyvinylamine membrane during the process of nanoparticle sediment and water evaporation. The membrane demonstrated CO_2_ permeance of 823 GPU and CO_2_/N_2_ selectivity of 242 at 0.5 MPa.

Ionic liquids (IL) as nonvolatile materials have received increasing interest in its applications for advanced gas separation membranes, including CO_2_ separation from N_2_ and methane. Recently, Bara et al. developed a new type of poly(IL)-IL membranes via the photopolymerization of acrylated IL monomer with free ILs trapped in the crosslinking network, and they achieved excellent CO_2_/CH_4_ selectivity of 119 at 20 °C [19]. It should be noted that the CO_2_ permeability of 20 Barrer limits its commercial application though the performance is above the 2008 Robeson Upper Bound. The membrane composition was further optimized including using a variety of free ILs with different cations and anions and the CO_2_ permeability increased to 40 Barrer [20]. An advantage of the application of non-amine IL membranes for flue gas carbon capture is the tolerance to contaminants such as sulfur oxides (SOx). By integrating compatible ionic liquids with poly(ionic liquids), membrane materials with synergistic properties can be obtained. Mittenthal et al. synthesized new ionic polyimides with amenability to thermal processing and film-forming ability. The ionic polyimides were able to spontaneously absorb ILs into their structures, indicating a potential orientation of the polymer chain and self-assembly of the ionic polymer and the ILs [21]. Further reducing the thickness is needed in the future to achieve high permeance. With respect to this point, Noble et al. developed the two-step coating technique for thin film fabrication, based on the property of poly(IL) to spontaneously absorb free ILs, as also found in the above-mentioned work of Mittenthal. With the thin film fabrication technique, they successfully achieved unprecedented CO_2_ permeance of 6000 GPU and CO_2_/N_2_ of 22 [22].

**Figure 2:**
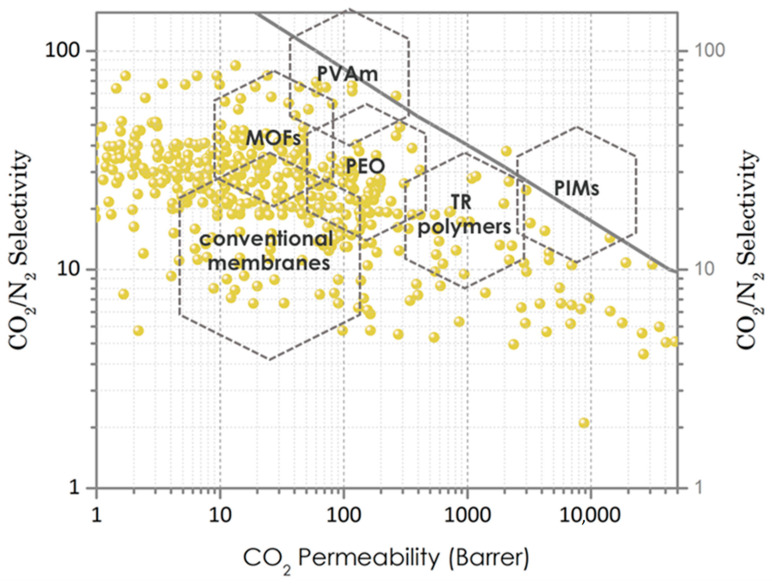
Robeson Upper Bound 2008 plot depicting CO_2_ permeability and CO_2_/N_2_ selectivity of gas separation membranes based on polyvinylamine PVAm, metal–organic frameworks (MOFs), polyethylene oxide (PEO), thermally rearranged (TR) polymers, polymers of intrinsic micro porosity (PIMs), and conventional membranes [15].

Polymers of intrinsic micro porosity (PIMs) are a new class of porous organic polymers (POPs), which possess high micropore and surface area compared to conventional polymers [23]. The polymer structure of PIMs consists of rigid and contorted monomers creating unprecedented micropores (0.7–1.1 nm) within the polymer chains. In general, the CO_2_ transport mechanism in PIMs depends on solution–diffusions, considering the pore size distribution. Accordingly, many studies have aimed to exploit these unique pores of PIMs to separate the CO_2_ as well as other gases such as nitrogen, hydrogen, methane, ethylene, etc. [24]. The gas separation performance of PIM membranes has been unprecedented compared to other rigid polymers, such as matrimid, and over 100 times higher CO_2_ permeability was achieved by PIMs. The Robeson Upper Bound plot, which depicts the trade-off between CO_2_ permeability and selectivity, has been well surpassed by numerous PIM-based membranes reported in the literature (Figure 2) [25]. For example, ultra-high CO_2_ permeable PIMs (PIM-TMN-Trip) have been reported by positioning triptycene in the backbone of the polymer [26]. The paddle-wheel-like structure of triptycene provides a free volume desired for high CO_2_ permeability.

Different types of bulky groups, including bridged-bicyclic ring systems and Troger’s Base, have also been considered to increase the free volume in PIMs. Polymers: PIM-EA-TB, PIM-TMN-SBI and PIM-Trip-TB showed very high CO_2_ permeability performance (>10,000 barrer) [27]. However, there are some drawbacks of PIMs that need to be addressed. First, the high CO_2_ permeability usually results in low to moderate selectivity over N_2_, CH_4_, and H_2_. In order to increase the selectivity, PIMs have been functionalized via polar groups such as tetrazole, triazine, amine, amide, etc. [28]. For example, Du et al. improved the CO_2_/N_2_ selectivity of PIM-1 over two folds by tetrazole functionalization (TZPIM) [29]. Nonetheless, the selectivity enhancement was accompanied by a great decrease in CO_2_ permeability in TZPIM, which is a typical case for membranes. Another strategy of boosting CO_2_ selectivity is to blend PIMs with highly CO_2_ selective polymers such as polyethylene glycol, matrimid, torlon, polysulfone, etc. The blending polymer usually exhibits higher CO_2_/N_2_ selectivity compared to PIMs. Similar to the functionalized PIMs, the selectivity enhancement has been reported with the following loss in CO_2_ permeability [5]. More recently, polyphosphazenes with ether side chains were blended with PIM-1 [30]. Improved CO_2_/N_2_ selectivity with high CO_2_ permeability was reported. Moreover, these blend membranes offered a solution to the brittle nature of PIM membrane films, which is another drawback reported for PIMs. The last major problem encountered in PIMs, as well as other glassy membranes, is aging [31]. The high CO_2_ permeability of freshly cast PIM membranes decreases dramatically over time. Structurally, PIMs yield a high free volume mostly from the inefficient packing of their polymer chains. Over time, the polymer chains relax; in other words, they reach the thermodynamic equilibrium that results in a great decrease in the free volume. Numerous studies have managed to impede the aging in PIM-based membranes. Aging-resistant membranes were fabricated by mixing or crosslinking PIMs with porous fillers such as MOFs and POPs [22,32]. Traditionally, adding a filler in membranes causes a selectivity drop in membranes, and most of the PIM-based mixed matrix membranes (MMMs) have followed this trend. However, several studies overcame the trade-off by maintaining high CO_2_ selectivity with significant CO_2_ permeability [3]. While some membranes achieved this by crosslinking [33], others added a third component (another polymer) to create blend polymer-based MMMs [34,35]. More importantly, some of these membranes showed great improvement in decreasing the aging. At this point, it should be noted that virtually all these membranes are thick films (<30 microns), and the aging phenomena in membranes should be investigated by considering practical applications, where membrane thickness is below one micron. It is well experienced that as the thickness of the membranes goes down, the aging in membranes accelerates. For example, PIM-1 was fabricated into a thick MMM by using a high surface area POP-type filler (PAF-1) [36]. Polymer chains of PIM-1 have been reported to be intercalated in the large pore of PAF-1, impeding the chain relaxation of PIM and thus the aging. However, Bakhtin et al. systematically compared the thickness effect in membranes and found that PIM/PAF-1 membranes also suffer from aging when fabricated in thin films [37].

Thermally rearranged (TR) polymers have emerged as another class of advanced gas separation membranes [38]. Following PIMs, TR polymers can also be categorized as a porous organic polymer given their high porosity [39]. The pores and free volume in TR polymers are created through thermal treatment: TR polymers are synthesized by the molecule rearrangement of polyimide and/or polyamides with ortho-functional groups (-OH, NH_2_ etc.) at high temperatures (400–600 °C). Consequently, a heterocyclic ring closure replaces imide and amide groups, creating more rigid polymers such as polybenzoxazoles and polybenzimidazoles. Similar to PIMs, rigid and bulky functional groups provide a free volume for CO_2_ molecules to permeate. In addition, heterocyclic rings such as benzoimidazole and benzoxazole bestow higher interaction with CO_2_ molecules compared to less polar gases [40]. Due to the microporous nature of TR polymers, the solution–diffusion mechanism directs the CO_2_ permeation. Hence, the conversion of polyimides into polybenzoxazole favors both the solubility and diffusivity of CO_2_ in the membranes. Recent studies have focused on further increasing this free volume in TR polymers. There are two major synthetic strategies that have been used: (i) introducing bulky functional groups into polymer chains, and (ii) crosslinking the polymer chains to adjust the pore opening and free volume. For example, TR polymers prepared from the crosslinking of polyimide with 1,4-butylene glycol evinced advanced CO_2_ permeability (8000 barer) with relatively high CO_2_/CH_4_ (17) selectivity. These reached high CO_2_/CH_4_ selectivity and at the same time maintained the high permeability rarely encountered in membranes. While TR polymers offer other desired membrane properties such as high chemical and thermal stability, resistance to plasticization, and tunable free volume, they also suffer from poor mechanical properties and competitive sorption between water and CO_2_. The high temperature used in TR polymer preparation rigidifies the polymer chains, and the membranes become very brittle. On the other hand, other polar gases such as water can compete with CO_2_ over interacting with the functional groups of TR polymers. This competition usually results in a CO_2_ permeability drop. Membranes fabricated from TR polymers also show aging when cast in thin films, following the fate of other porous polymeric membranes. Unlike PIMs, MMM fabrication based on TR polymers is very difficult, because the thermally crosslinked polymer chains are not soluble in common solvents.

Metal–organic frameworks have been a frontrunner material to be used as a filler particle in mixed matrix membranes. MOFs consist of crystalline two and three-dimensional structures with permanent porosity and unprecedented surface area. Pores of MOFs are easily adjustable and functionable, which make them a strong candidate to be used in various gas separation and gas capture applications. More recently, MOFs have been also considered as a gas separation membrane [41]. Pure MOF membranes have been fabricated on a support by several methods including direct growth, secondary growth using MOF seeds, and in situ growth [42]. In general, the pore-sized distribution of MOFs is in the microporous region (<2 nm). Therefore, the CO_2_ transport mechanism relies mostly on the solubility of CO_2_, which is similar to PIM and TR polymer-based membranes. Therefore, the pore structure of MOFs has been adjusted to increase CO_2_ permeability. A well-known class of MOFs, zeolitic imidazolium frameworks (ZIFs), has been the most studied MOF for the fabrication of pure MOF membranes. The main reason is the facile synthesis of ZIFs under mild conditions (room temperature, water solvent, etc.). For example, ZIF-8 membranes grown on hollow fibers (ZIF-8-HF) showed high CO_2_/N_2_ selectivity (52) [43]. However, these membranes suffered from low CO_2_ permeabilities along with other ZIF-based MOF membranes. The continuous flow membrane fabrication on a fiber showed the feasibility of large-scale production, which is usually a hurdle for MOF membrane fabrication. There are also other types of MOFs such as HKUST-1 and SIFSIX-3-Ni, which exceeded the CO_2_ permeability of ZIF membranes [44]. MOF membranes stand as an advanced gas separation membrane candidate. However, there is still big room to improve MOF membranes, as they mostly suffer from the trade-off between CO_2_ permeability and selectivity. In addition, other important membrane properties such as mechanical strength, MOF penetration into the support, and defects should be taken into account.

In addition to the glassy polymers mentioned so far, several new high-performance membranes have been also fabricated by using rubbery polymers. Remarkably, rubbery polymers do not suffer the physical aging in thin films that commonly exist in glassy polymers. The high flexibility of the rubbery polymer chains accounts for the large gas diffusivity. Ether oxygen has a strong affinity to CO_2_, endowing the ether oxygen-containing rubbery material with great potential to surpass the Robeson Upper Bound. In 2019, a group of ether oxygen-containing oligomers with the O/C ratio of 0.71, which is much greater than the 0.5 in polyethylene oxide, was reported by Lin’s group of the University of Buffalo [45]. This work has enlightened a new direction of molecular design for CO_2_-philic rubbery membranes. Another type of emerging rubbery polymers used in CO_2_ separation is polyphosphazenes [46]. Polyphosphazenes consist of a phosphazene backbone, which provides both flexibility and tunable chemical structure by the substitution of CO_2_ attractive groups such as ether. More importantly, polyphosphazene membranes do not show crystal domains as in the other ether-containing polymers, such as polyethylene glycol, which limit the CO_2_ permeability. Although these polymers have been offered for gas separation before, it was only very recently that high CO_2_ separation performance was achieved by crosslinking [47] and blending with PIMs [30].

## 3. Conclusions

Membrane technology has become a competitive alternative technology for carbon capture to the conventional amine scrubbing technology. Recent developments in facilitated transport, porous polymer, MOF-based and PEO-based membranes demonstrated great potential for membrane technology to achieve highly energy-efficient and cost-effective carbon capture. Future challenges to improve the membrane-based carbon capture lie in the thin film composite (TFC) membrane and its module fabrication as well as rational process design. The MOF-based membrane in particular faces challenges in TFC fabrication and membrane scaling up, including MOF material scaling up. Some successful TFC examples have been discussed in this review. More TFC study is expected to convert the high permeability of MOF-based bulk membranes into the excellent permeance of TFC in the near future. A rational process design is helpful to fully leverage the performance characteristics of different membrane systems. For instance, a facilitated transport membrane process does not need a moisture removal process prior to the membrane separator and is more suitable with permeate vacuum instead of air sweep. Last but not the least, in the application of carbon capture, the presence of contaminants should also be taken into consideration. In this regard, field testing should be included as an important part of the development loop of membrane technology.
